# When Cognitive Reflection Leads to Less Overall but More Systematic Judgment Bias: The Case of the Base Rates Fallacy

**DOI:** 10.3390/jintelligence11060100

**Published:** 2023-05-24

**Authors:** Mário B. Ferreira, Hugo Assunção, Amanda Seruti

**Affiliations:** CICPSI, Faculdade de Psicologia, Universidade de Lisboa, 1649-013 Lisboa, Portugal

**Keywords:** base-rate problems, heuristics, cognitive reflection, bias, noise

## Abstract

Although widely used in the judgment under uncertainty literature, the so-called Lawyer–Engineer problem does not have a Bayesian solution because the base rates typically oppose qualitative stereotypical information, which has an undefined diagnostic value. We propose an experimental paradigm that elicits participants’ subjective estimates of the diagnosticity of stereotypical information and allows us to investigate the degree to which participants are able to integrate both sources of information (base rates and stereotypical descriptions) according to the Bayesian rule. This paradigm was used to test the hypothesis that the responses (probability estimates) to the Lawyer–Engineer problem from more rational individuals deviate from normative Bayesian solutions in a way that shows smaller but more systematic bias. The results further suggest that the estimates of less rational participants are noisier (less reliable) but may be more accurate when aggregated across several problems.

“To understand error in judgment we must understand both bias and noise”(Kahneman et al. 2021, p. 5).

## 1. Introduction

Research in the realm of judgment under uncertainty has shown that people tend to infringe even the most elementary logical or probabilistic rules in reasoning tasks that strongly cue intuiOppenheimertive responses different from the ones stemming from these rules (e.g., De Neys and Pennycook 2019; Evans 2016; Gilovich et al. 2002; Kahneman 2003; Stanovich and West 2000).

One domain in which human judgment often departs from normative rules of probability is belief updating. Specifically, when updating their beliefs, reasoners should integrate the prior probabilities of the event into question with acquired (independent) evidence, as prescribed by Bayes’ theorem. However, reasoners often fail to appreciate the relevance of prior probabilities (e.g., Bar-Hillel 1980). This failure was dubbed the base-rate fallacy, and it has been studied using different types of base-rate problems (Barbey and Sloman 2007; Fong et al. 1986; Gigerenzer and Hoffrage 1995; Kahneman and Tversky 1973; Pennycook and Thompson 2012).

One of the most studied base-rate problems is the so-called Lawyer–Engineer (L–E) problem (Kahneman and Tversky 1973). L–E problems typically present a description of a target individual randomly drawn from a sample including people from two social groups of different sizes (e.g., 30 engineers and 70 lawyers). The description is stereotypical of the smaller group (e.g., “this person enjoys reading science fiction and writing code on their computer”).

After reading the problem, participants are asked to identify to which group is the target more likely to belong or to estimate the probability that the target individual is a member of the larger (or smaller) group.

Notably, L–E problems do not have a Bayesian solution because the diagnostic value of the stereotypical description is undefined. Although previous work has addressed this point (e.g., Politzer and Macchi 2005), no research, to the best of our knowledge, has proposed a way to find the exact Bayesian solution to this type of base-rate problem.

To overcome this limitation, in this study, we asked participants to estimate the percentage of people in the two groups (e.g., engineers and lawyers) that fit the stereotypical description presented in the problem (e.g., “this person enjoys reading science fiction and writing code on their computer”). After estimating these percentages, participants were then presented with the L–E problem.

This strategy allowed us to compute the perceived diagnosticity of the stereotypical information for each participant and to thereby assess the extent to which participants’ responses deviated from the Bayesian solutions. We hypothesized that response bias (i.e., the deviation from the Bayesian solution) would be predicted by individual differences in rational thinking as measured by using the cognitive reflection test (Frederick 2005). Specifically, more rational participants were predicted to show smaller but more systematic (less noisy) deviations from the Bayesian solution, which would translate into less overall bias but more systematic bias.

## 2. Previous Research on the L–E Problem

Initial research with the L–E problem showed that participants responded according to the diagnostic value of the provided stereotypical description, apparently neglecting the base rates (e.g., Kahneman and Tversky 1973). This was taken as an illustration of the representativeness heuristic (Kahneman and Tversky 1972), according to which, people tend to ignore the base rates in favor of the individuating information because they equate the original question, “To which group is the target more likely to belong?” with the simpler question, “How similar is the description to the stereotypes of the groups?” (Kahneman and Frederick 2002).

Other accounts of base rate negligence include the probability mental model account (e.g., Gigerenzer 1991; Gigerenzer et al. 1991) and the pragmatic approach to thinking and reasoning (e.g., Hilton 1995; Macchi 1995, 2000; Politzer and Macchi 2005). The first argues that the L–E problem activates an inferential framework or “mental model” to solve the problem that usually does not take into consideration the random sampling of the target individuals. Indeed, in studies where the random sampling is shown to or performed by the participants, the base-rate neglect is considerably reduced (Gigerenzer et al. 1988; Nisbett and Ross 1980).

The second (pragmatic approach) readdresses the origins of many reasoning biases and errors by carefully analyzing potential discrepancies between the representation of the task that participants are likely to build and what the experimenter aims to convey. To illustrate, Schwarz et al. (1991) showed that participants relied more on the individuating information in the L–E problem when descriptions were presented as a profile written by a psychologist rather than a piece of information formulated by a computer. According to the authors, this indicates that the participants’ use of information depends on the communicative intentions of the experimenter.

Subsequent research aimed at testing these and other accounts has demonstrated that several other factors may decrease people’s tendency to neglect base-rate information. For instance, base rates are more likely to be considered when this statistical information is perceived to be causally relevant (Ajzen 1977); when a clear causal link for stereotypes is provided (Turpin et al. 2020); when it is learned from experience (Betsch et al. 1998); when it varies within participants (Fischhoff and Bar-Hillel 1984); when it is derived from a representative sample (Wells and Harvey 1977); when the description (individuating information) lacks diagnostic value (Ginossar and Trope 1987).

These studies triggered an interesting debate over whether people are able to reason in a Bayesian way (see Koehler 1996). Taken together, research focusing on the L–E problem seemed to converge on the view that although base-rate information is rarely fully ignored, it is usually insufficiently considered in reasoners’ judgments (Benjamin et al. 2019; Pennycook and Thompson 2016).

However, the L–E problem does not have a Bayesian solution. There is no unique normative response as to how participants should integrate base rates with the descriptive stereotypical information because that depends on the diagnostic value of the stereotypical evidence, which is undefined.

Furthermore, Pennycook and Thompson (2012; see also Politzer and Macchi 2005) showed that responses (probability estimates) to these problems typically cluster in one of two extremes. They are consistent with the stereotypical information (e.g., “this person is an Engineer”) and interpreted as instances of base-rate neglect, or they correspond to the base rates (e.g., “this person is a Lawyer”), which are often interpreted, perhaps too hastily, as the rational response. In fact, from a Bayesian perspective, both of these answers are irrational because both show a failure to integrate base rates with descriptive information (Pennycook and Thompson 2016).

Our goal in this paper is twofold. First, we propose a new experimental paradigm that allows us to investigate the degree to which participants are able to integrate the base rates and the stereotypical description in the L–E problem according to the Bayesian theorem. Second, we tested the hypothesis that the probability estimates of more rational individuals (as measured with the cognitive reflection test; Frederick 2005) deviate from normative Bayesian responses in a way that shows less overall bias but more systematic bias.

## 3. Bayesian Solutions to the Lawyer–Engineer Problem

To achieve this goal, one needs to know in the first place how well the target descriptive information presented in the problem discriminates between the two groups, or in other words, how diagnostic the description is perceived to be by the reasoner. Because this is a subjective estimate, different reasoners will differ in the way they evaluate the diagnosticity of the same descriptive information. Thus, different responses to the same base-rate problem may be considered correct as long as they are internally consistent, that is to say, as long as they stem from the appropriate (Bayesian) integration of the base rates and the perceived diagnosticity of the descriptive information.

In order to examine reasoners’ ability to integrate base rates with the provided descriptive (stereotypical) information, we requested that participants estimate the percentage of members of the minority group and members of the majority group that fit the description. This was done right before they answered the corresponding base-rates problem. The elicitation of these estimates allowed us to compute the subjective diagnostic value of the description and to compare responses to the problems to a Bayesian normative standard (see Method section for details).

To analyze participants’ answers to the base-rate problems, two dependent measures were considered: (a) the mean deviation from the Bayesian response or mean error, and (b) the mean absolute deviation from the Bayesian response or mean absolute error.

In the first case, positive and negative deviations from the Bayesian solution across the problems tend to cancel each other out (i.e., if the answer to a problem is two points below the correct response and the answer to another problem is two points above, then the participant’s mean error would be zero: (−2 + 2)/2 = 0). Hence, the mean error will be greater for participants who show a directional or systematic bias in their responses to the problems (otherwise, errors will tend to cancel each other out).

In the second case, deviations are considered in module (absolute value), and thus, they do not cancel each other out across problems (i.e., if an answer is two points below the correct response and another answer is two points above, the mean absolute error would be two: (|−2| + |2|)/2 = 2). As a result, the mean absolute error will increase as deviations from the Bayesian solution increase, regardless of the direction of these deviations. In sum, the mean error is a measure of participants’ systematic bias, whereas the mean absolute error measures participants’ overall bias.

According to the dual-process framework of cognitive higher processes (Evans and Stanovich 2013), the deliberate integration of statistical and descriptive information requires cognitively demanding Type 2 processing. Because reasoners usually forego effortful Type 2 processing in favor of Type 1 responses that quickly come to mind, the latter responses are naturally preferred over deliberate and effortful integration of information (Kahneman 2003; Kahneman and Frederick 2002; Stanovich and West 2000). However, some reasoners seem to have the disposition and cognitive capacity to (successfully) override cognitive miserliness, that is, to more often second-guess their first intuitions and to engage in effortful deliberation.

It follows that individual differences in rational thinking (Stanovich 2009) are a likely predictor of response bias to the base-rate problems used in the present study. With this in mind, participants were requested to respond to an extended version of the Cognitive Reflection Test (Frederick 2005).

The Cognitive Reflection Test is a widely used measure of the degree to which individuals override an intuitive response and engage in reflection, and it has been shown to be a predictor of performance on tasks from the heuristics and biases literature (Toplak et al. 2011). As such, we expected that reasoners higher in cognitive reflection would show less bias in terms of mean absolute error (i.e., overall bias). In other words, mean responses would deviate less from correct Bayesian responses as the level of cognitive reflection increased.

However, we further hypothesized that reasoners higher in cognitive reflection would tend to show a greater response bias in terms of mean errors. This was predicted to be so because these reasoners were expected to address the base-rate problems in a more systematic way, that is, their response bias was expected to be smaller but more directional (i.e., less noisy). The aggregation of these minor but more systematic deviations from the normatively correct answer reduces the chances of bias cancellation, and therefore, it is likely to lead to increased mean error.

The successful override of cognitive miserliness as measured using the CRT involves a combination of both cognitive ability and epistemic disposition to engage in effortful reasoning. Thinking dispositions alone (as measured with self-report questionnaires) and cognitive ability (as measured with fluid intelligence tests) are often seen as means to rationality but not sufficient conditions in themselves for rational reasoning (Stanovich 2009, 2016; Stanovich and West 2008).

In order to assess the effect of thinking dispositions on performance, participants completed the rational–experiential inventory (REI; Epstein et al. 1996; Pacini and Epstein 1999). The REI includes an adapted version of the Need for Cognition (NFC) scale, which measures the degree to which one engages in and enjoys thinking (Cacioppo and Petty 1982; Cohen 1957), and a Faith in Intuition (FI) scale, which measures the tendency to rely on one’s intuition.

To assess the impact of cognitive ability (fluid intelligence), participants responded to a short version of the Raven’s Progressive Matrices (RPM) test, which has been shown to have psychometric properties comparable to those of the full-length Raven’s Progressive Matrices test (Bilker et al. 2012).

## 4. Experiment Overview

Participants answered six L–E base-rate problems (BRP). For each BRP, participants were asked to estimate P(A/C), the probability that a target (randomly chosen from a sample of members of groups A and B) belongs to the smaller group A given that the target has characteristics C (stereotypical of group A).

The Bayesian solutions to the BRP used are unspecified, as they are dependent on the diagnosticity of the stereotype-based descriptions. These, in turn, depend on the unknown probabilities P(C/A) and P(C/B). Hence, before responding to the problems, each participant was asked to estimate these conditional probabilities (e.g., “estimate the percentage of Engineers/Lawyers that enjoys reading science fiction and writing code on their computer”).

The base rates presented in each problem varied for each participant depending on the participant’s perceived diagnosticity of the description in such a way that the correct Bayesian answer was the same across participants. Specifically, the Bayesian solution was P(A/C) = 0.25 for two of the BRP; P(A/C) = 0.50 for two other BRP; P(A/C) = 0.75 for the remaining two BRP.

For each participant, we computed two dependent measures: the mean error (i.e., mean deviation from the Bayesian responses) and the mean absolute error (mean absolute deviation from the Bayesian response). As aforementioned, higher scores in the CRT were predicted to be associated with less overall bias but more systematic bias.

## 5. Method

Participants. Eighty participants (30 males; M_age_ = 33.82, *SD* = 18.46) participated in this experiment in a study wave for a credit course. The sample was composed of students from the University of Lisbon. The study was approved by the ethics committee of the Faculdade de Psicologia, Universidade de Lisboa.

Material. Participants answered a questionnaire including 6 Base-Rate Problems (adapted from De Neys and Glumicic 2008), 6 Cognitive Reflection Test Problems (adapted from Frederick 2005; Thomson and Oppenheimer 2016), 9 Raven’s Progressive Matrices problems (Bilker et al. 2012), and 10 items (a 5-items NFC scale and a 5-items FI scale) from the short version of the Rational-Experiential Inventory (Norris et al. 1998) (see Appendix A).

Each Base-Rate Problem (BRP) presented a sample of 100 individuals divided into a minority group A and a majority group B. The number of people in each of the two groups (base rates) was presented in frequencies. In each BRP, a target person, randomly drawn from the sample, was described as having characteristics C, which are stereotypical of group A.

Procedure. In order to obtain the P(C/A) and P(C/B) for each BRP, in the beginning of each experimental trial, participants were asked to estimate the percentage of members of group A (e.g., Engineers) and the percentage of members of group B (e.g., Lawyers) with characteristics C on a 100-point scale from 0% to 100%. For example:

- What percentage of engineers enjoys reading science fiction and writing code on their computer?
- What percentage of lawyers enjoys reading science fiction and writing code on their computer?

After providing these two estimates, the corresponding BRP was presented.

Based on the participants’ estimates of P(C/A) and P(C/B), the Bayesian normative solution could be calculated for each participant response to each BRP using the Bayes theorem:

$$P(A|C)=\frac{P\left(A\right)\times P(C|A)}{P\left(A\right)\times P\left(C|A\right)+P\left(B\right)\times P(C|B)}$$

where P(A) and P(B) represent the prior probabilities (base rates).

In order to make the normative Bayesian solutions converge on the same responses across participants, the base rates presented in each BRP were computed separately for each participant using the following derivation of the Bayes theorem:

$$P(A)=\frac{P\left(C|B\right)\times P(A|C)}{P\left(C|A\right)-P\left(C|A\right)\times P\left(A|C\right)+P\left(C|B\right)\times P(A|C)}$$

In this way, the Bayesian solutions were specified to be P(A/C) = 0.25 for one-third of the BRP, P(A/C) = 0.50 for the other third, and P(A/C) = 0.75 for the remaining third.^1^ The conditions were counterbalanced so that each participant received two problems of each kind in random order.

To illustrate, if a participant estimated that 90% of the Engineers and 30% of the Lawyers fit the description (i.e., “enjoys reading science fiction and writing code on their computer”), then, for a Bayesian solution of P(A/C) = 0.25, the corresponding base rates would be 10 Engineers and 90 Lawyers, and the BRP presented to this participant would be:

In a study, 10 Engineers and 90 Lawyers were interviewed.

One of these 100 persons was randomly selected.

This person enjoys reading science fiction and writing code on their computer.

Remember that according to your opinion, 90% of the Engineers and 30% of the Lawyers enjoy reading science fiction and writing code on their computer.

What’s the probability that this person is an Engineer?

Participants responded to each BRP on a 100-point scale from 0% to 100%. In sum, across six trials, the task of the participants was to first estimate the percentages of members of group A and group B with characteristics C and then to respond to the corresponding BRP in which they were asked to estimate the likelihood, in percentage, that a target person chosen at random would belong to group A. While responding to the corresponding BR problem, each participant was reminded of their own P(C/A) and P(C/B) estimates. Before beginning the experimental trials, participants had one practice trial to better understand the structure of the task and clarify any doubts with the experimenter.

The mean diagnosticity of the descriptions included in the six BRP varied between 51% and 73% (see Appendix A). This means that, on average, BRPs with the Bayesian solution P(A/C) = 0.25 were conflict problems, that is, problems where the presented base rates must oppose (be in conflict with) the diagnosticity of the information in order to make the Bayesian solution converge on 25%. In contrast, BRPs with the Bayesian solution P(A/C) = 0.75 were no-conflict problems, or problems where the base rates must be in the same direction of the diagnosticity of the information in order to make the Bayesian solution converge on 75%.^2^

The main dependent variables included two accuracy measures:

Response Error=Answer to the BRP−Bayesian Solution

Response Absolute Error=|Answer to the BRP−Bayesian Solution|

Mean errors and mean absolute errors were computed for each participant across the 6 BRP.

## 6. Results and Discussion

The mean error to the BRP was −4.48% (*SD* = 27.67%), and the mean absolute error was 20.28% (*SD* = 19.33%).

**Figure 1:**
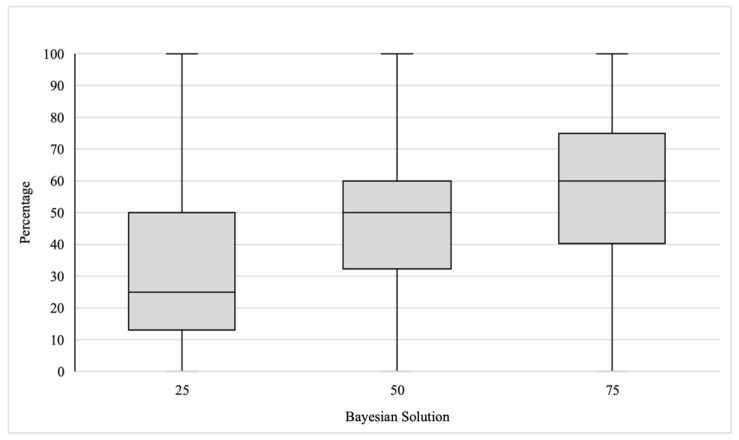
Mean, median, and response quartiles of the perceived probability that the target individual belongs to the smaller group for each of the specified Bayesian solutions.

A one-way ANOVA with responses to the BRP as the dependent variable showed the main effect of the Bayesian solution experimental condition ((25%, 50%, 75%), *F*(2,477) = 32.47, *p* < .001, η2 = 0.120). Mean responses (see Table 1, first column) were close to the BRP Bayesian solutions. Median responses actually corresponded to the Bayesian solutions for the 25% and 50% conditions, and for the 75% condition, the median response was equal to 60% (see Figure 1).

These results indicate that participants were able to combine both sources of information when responding to the BRP and that overall, they were quite sensitive to the base rates as they integrated this information with the perceived diagnosticity of the descriptions.

However, mean estimates deviated from the Bayesian solutions. A one-way ANOVA with mean response errors as the dependent variable showed the main effect of the Bayesian solution experimental condition ((25%, 50%, 75%), *F*(2,477) = 47.02, *p* < .001, η2 = 0.165). As displayed in Table 1 (column 4), mean deviation was positive for the 25% condition (i.e., conflict problems), mildly negative for the 50% condition, and negative for the 75% condition (i.e., no-conflict problems). Note that both responding above the Bayesian solution for conflict problems (where the base rates opposed the description) and responding below the Bayesian solution in no-conflict problems (where the base rates agree with the descriptive information) indicate that participants tended to underweight the base rates. Interestingly, the base-rate neglect was higher for no-conflict problems than for conflict problems. This suggests that integrating the base rates with the description in a way that is closer to the prescribed Bayes rule is easier when the two sources of information oppose each other than when they point in the same direction.

Mean absolute estimates also deviated from the Bayesian solutions (Table 1, Column 5) but did not show a clear trend across conditions. A one-way ANOVA with mean absolute error as the dependent variable did not reach significance (*F*(2,477) = 2.95, *p* = .053, η2 = 0.012).

## 7. Assessing the Effect of Thinking Dispositions and Cognitive Ability on Performance

Participants’ CRT scores are positively associated with measures of fluid intelligence (MPR) and Need for Cognition (NFC). In other words, a disposition to enjoy thinking, fluid intelligence, and the successful override of cognitive miserliness share some variability.

When looking at performance measures, the mean error only correlates with the CRT, whereas the mean absolute error correlates with both the CRT and MPR (see Table 2).

To assess the effect of thinking dispositions and cognitive ability on performance, the CRT, MPR, NFC, and FI scores were entered in a multiple regression analysis as predictors of participants’ mean errors. The mean error was only predicted with CRT scores (β = −0.26, *p* = .03). No other predictor reached statistical significance (see Table 3).

**Figure 2:**
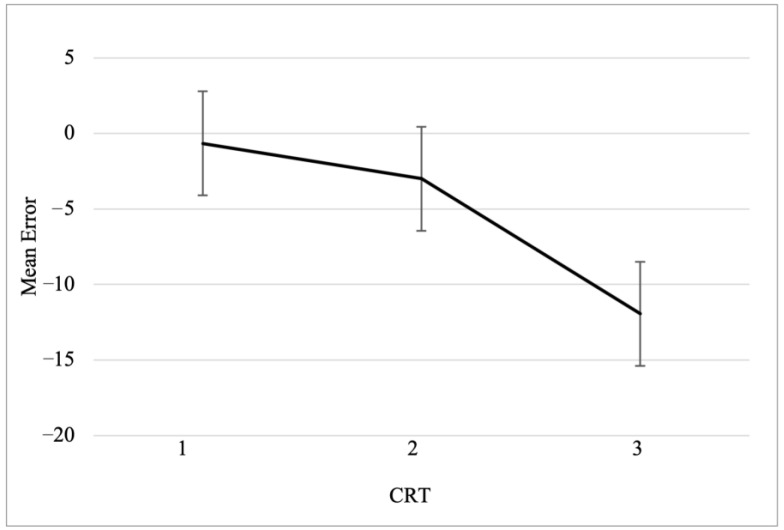
Mean error as function of cognitive reflection. CRT scores were categorized in three groups: low (zero or one correct response), intermediate (two, three, or four correct responses), and high (five or six correct responses).

In order to display the effect of cognitive reflection on mean error, participants were categorized in three groups: low (zero or one correct response), intermediate (two, three, or four correct responses), and high (five or six correct responses) cognitive reflection (see Figure 2). Participants low in cognitive reflection had a mean error close to zero (*M_low_* = −0.66), and the mean error progressively increased for participants with intermediate (*M_intermediate_* = −3) and high levels (*M_high_* = −11.9) of cognitive reflection. This increased bias in mean error has a negative sign, which suggests a progressively increasing tendency to underestimate base rates.

The same scores (CRT, MPR, NFC, and FI) were also entered in a multiple regression analysis as predictors of participants’ absolute mean errors. Performance in the CRT (β = −0.24, *p* = .04) and NFC (β = 0.31, *p* = .005), were both significant predictors of absolute mean error (see Table 4).

**Figure 3:**
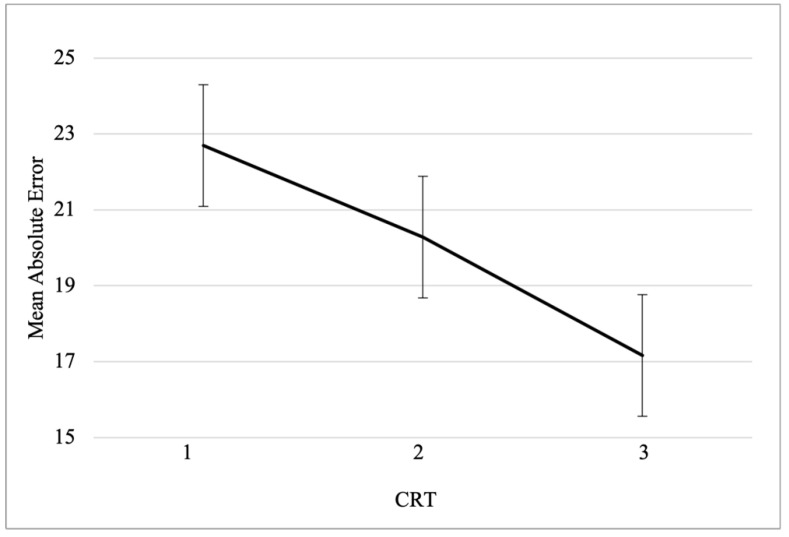
Mean absolute error as function of cognitive reflection. CRT scores were categorized in three groups: low (zero or one correct response), intermediate (two, three, or four correct responses), and high (five or six correct responses) cognitive reflection.

As displayed in Figure 3, participants with low, intermediate, and high levels of cognitive reflection showed a progressive decrease in mean absolute error (*M_CRT_low_* = 22.69; *M_CRT_intermediate_* = 20.28; *M_CRT_high_* = 17.16).

**Figure 4:**
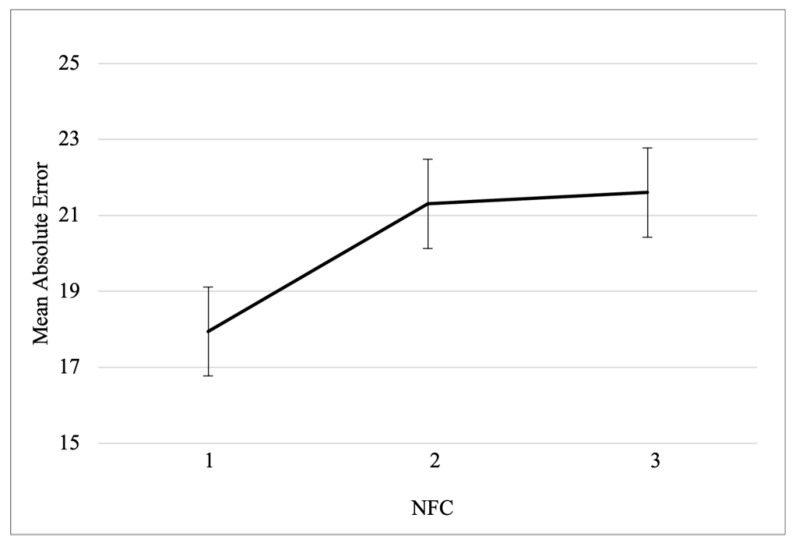
Mean absolute error as function of Need for Cognition scores. NFC scores were categorized in terciles: low (1st tercile), intermediate (2nd tercile), and high (3rd tercile) needs for cognition.

In order to display the impact of NFC on absolute mean error, NFC scores were categorized in terciles: low, intermediate, and high needs for cognition. As shown in Figure 4, mean absolute error increased as function of NFC (*M_NFC_low_* = 17.94; *M_NFC_intermediate_* = 21.3; *M_NFC_high_* = 21.6).

In sum, when controlling for performance in the CRT as well as the remaining predictors (FI and RPM) people with higher needs for cognition actually showed more overall biases. Apparently, an individual tendency to enjoy thinking (Cacioppo and Petty 1982) may promote even greater biases when people’s actual ability to successfully detect and override cognitive miserliness (as measured with the CRT) is controlled for.

## 8. General Discussion

In the present study, how reasoners integrate the prior probabilities of an event with acquired descriptive evidence was investigated using a classic base-rate neglect task of the heuristics and biases research tradition: the L–E problem (Kahneman and Tversky 1973).

This problem typically provides two sources of information: the base rates (the relative size of two social groups, a smaller group A and a larger group B) and a description of a randomly sampled target person that is stereotypical of the smaller group. To give a probabilistic correct answer to the question, “How likely it is that the target person belongs to group A?” one must integrate both sources of information into a single likelihood estimate using the Bayes rule.

Previous research using the L–E problem has shown that people often neglect or underweight the base rates when other stereotypical (more intuitive) sources of information are available (e.g., De Neys 2012; De Neys and Glumicic 2008; Kahneman and Tversky 1973; Nisbett and Borgida 1975; Pennycook et al. 2015; Pennycook and Thompson 2016). However, in this study, the stereotypical description is qualitative in nature, and thus, its diagnostic value is undefined. To overcome this limitation in this study, we asked participants to estimate the likelihood of occurrence of the description in both social groups before they answered to the corresponding BRP. This allowed us to compute how diagnostic the descriptions were perceived to be by each participant.

Based on the elicited perceived likelihoods of occurrence, each participant then responded to the BRP with the base rates adjusted in such a way that the normative Bayesian solution could be standardized across participants ((P(A/C) = 25%, 50%, or 75%).

In this way, it was possible to investigate people’s ability to appropriately integrate the base rates with the target’s descriptive information. The results indicate that participants not only did not neglect the base rates, but they were also quite sensitive to them (and at some points quite close to the Bayesian solutions) in the way they responded to the problems.

This overall sensitivity to base rates might seem to contradict extant research showing a tendency to neglect base rates. In this respect, it may be important to note that in the present task, each L–E problem presented a different question together with a base rate that also varied across problems (and participants). This contrasts with the original work of Kahneman and Tversky (1973), which presented to participants just one base rate with five varying descriptions. These experimental differences certainly have an impact on the consideration of the base rates. Fischhoff et al. (1979) showed that when the base rate was varied within participants, the performance more often reflected the base rates. Since in our study both base rates and (estimated) diagnosticity varied within participants, performance results may have more often reflected both sources of information.

However, the mean deviation from the Bayesian solutions was consistent with a tendency to underweight the base rates. This happened mostly when there was no conflict between the base rates and the descriptive information (i.e., for the BRP with a Bayesian solution of 75%).

This propensity is interesting because no-conflict problems have been used as the baseline in studies in which participants are asked to make dichotomous choices (exactly because descriptions and base rates converge in the same answer, e.g., Ferreira et al. 2006). However, our experimental approach suggests that people may have more difficulty adequately integrating base rates with the descriptive information in no-conflict problems than when these sources of information are in opposition (conflict problems). This does not put into question the research logic of contrasting performance in no-conflict and conflict problems. It does, however, indicate that merely relying on data obtained via dichotomous choices paradigms may mask relevant findings concerning how people integrate information and make judgments under uncertainty.

Specifically, less sensitivity to base-rate information when base rates point in the same direction of the descriptive information suggests that the way people integrate information was not Bayesian but perhaps better described as the computation of an average (between both sources of information). That would explain why participants’ estimates are close to the Bayesian solutions when there was opposition between the two sources of information (but not when both sources showed convergence).

As predicted, more rational people showed a reduced overall bias, that is, people who are better at overriding their initial intuitions and engaging in reflection were also better at integrating the diagnostic value of the descriptive information with the base rates (Bonner and Newell 2010; Kahneman 2003; Ferreira et al. 2016).

However, the more rational reasoners tended to show more systematic bias (i.e., they deviated from the normative Bayesian solutions in a more regular or directional way). This contrasts with responses from less rational people, which were more biased overall and more unreliable in their estimates. Ironically, less reliable (noisier) estimates allowed for error cancelation, which may explain the reduced systematic bias of less rational people.

Difficulties in integrating the diagnostic value of the descriptive information and the base rates is one factor that might have contributed to the results pattern of less rational people. Their responses (probability estimates) to the BRP have been shown to be consistent with only one piece of information: the perceived diagnosticity of the description or the base rates (Evans and Elqayam 2007; Pennycook and Thompson 2012). In the present study, such estimates often deviated from the Bayesian solutions in opposite directions, eventually leading to a decreased mean error (and increased absolute error). Attention capturers (Sanford et al. 2006), which serve to highlight certain parts of an utterance so that they are attended to in greater detail and processed in greater depth (written text examples include underlining, italics, and boldface), could be used to experimentally examine the aforementioned explanation by testing the effects of drawing reasoners attention to one (base rates) or the other (diagnostic value of description) or both sources of the information in BRP.

One limitation of the present study concerns the discrepancy between the way the conditional probabilities, P(C/A) and P(C/B), and the responses to the BRP, P(A/C), were elicited. Participants estimated the first using a relative frequencies format (e.g., “What percentage of engineers/lawyers enjoys reading science fiction and writing code on their computer?”), whereas they answered the BRP using a single case format (e.g., “What is the probability that this person is an engineer?”). The use of relative frequencies has been shown to lead to improved accuracy in judgment under uncertainty (Cosmides and Tooby 1996; Fiedler 1988; Hoffrage et al. 2002; Gigerenzer and Hoffrage 1995, but see Sloman et al. 2003; Sprenger and Dougherty 2006). Hence, future research should systematically vary the two formats in order to investigate their impact on participants’ ability to integrate both sources of information (diagnostic and base-rate information).

Furthermore, the extremity of the base rates presented to participants, which have been shown to affect participants’ (implicit) responses to the BRP (e.g., Ricco et al. 2023), were not systematically manipulated in the present study. However, we provide an experimental paradigm that may be used in future research to study these and other variables in a more refined way.

## 9. Conclusions

Recently, Kahneman, Sibony, and Sunstein argued that “to understand error in judgment we must understand both bias and noise” (Kahneman et al. 2021, p. 5). They further argued that systematic bias has so far been the star of the show and that the importance of noise, as a source of judgment error, has rarely been recognized.

Here, we predicted and found that deviations from normative Bayesian responses (mean error) were higher among more rational participants (as measured with the CRT) due to a minor but systematic underweighting of base-rate information.

These findings may appear to be at odds with the widespread notion that bias is the result of Type 1 processes when Type 2 reasoning fails to override wrong (type 1) intuitions and engage in hypothetical reasoning (e.g., Stanovich 2016). However, we would like to argue that our findings actually expand (and do not contradict) this notion. Indeed, given the more systematic nature of Type 2 reasoning, and as long as these judgments under uncertainty are not fully error-free (Ferreira et al. 2022), the accumulation of smaller but more systematic Type 2 deviations from the normative correct responses may naturally lead to increased mean error. In other words, bias in judgment under uncertainty may stem from Type 1 as well as Type 2 processing.

Our findings further suggest that less rational participants may make more accurate mean judgments when responding to several L–E problems. This seems to be the case because response error is larger but also more variable (above and below the normative correct answer) for these participants, which allows for error cancelation across trials.

In sum, Kahneman et al. (2021) are certainly right when they call attention to the perils of noise as a major source of judgment flaws. However, the silver lining to noise might be that at least in certain conditions (e.g., when the decision is based on the accumulation of several similar judgments), one may be “noisier” but not necessarily more biased in their judgments under uncertainty.

## Figures and Tables

**Table 1:** Mean responses, median responses, mean error, and mean absolute error by experimental condition (i.e., Bayesian solutions 25%, 50%, and 75%).

Bayesian Solution	Mean Response	Median Response	Mean Error	Mean Absolute Error
25%	33.3% (27%)	25%	8.3% (27%)	20.6% (19.2%)
50%	47.1% (24%)	50%	−2.8% (24%)	17.5% (16.7%)
75%	56.0% (24.8%)	60%	−18.9% (24.8%)	22.7% (21.4%)
Total	45.5% (26.9%)	50%	−4.4% (27.6%)	20.2% (19.3%)

**Table 2:** Correlation (Pearson r) among mean error (ME), mean absolute error (MAE), Cognitive Reflection Test (CRT), Raven’s Progressive Matrices (MPR), Need for Cognition (NFC), and Faith in Intuition (FI).

	CRT	MPR	NFC	FI
ME	−0.253 *			
MAE	−0.230 *	−0.230 *		
CRT		0.376 ***	0.330 **	
MPR	0.376 ***			
NFC	0.330 **			
FI				

*** < .001/** < .01/* < .05.

**Table 3:** Multiple linear regression results with Cognitive Reflection Test (CRT), Raven’s Progressive Matrices (MPR), Need for Cognition (NFC), and Faith in Intuition (FI) as predictors and participants’ mean error as the criterion.

	B	SE B	β	*T*	*p*
CRT	−2.30	1.09	−0.26	−2.10	0.03 *
MPR	0.15	0.94	0.01	0.16	0.87
NFC	0.32	0.57	0.06	0.55	0.57
FI	0.42	0.58	0.08	0.72	0.47

* < .05.

**Table 4:** Multiple linear regression results with Cognitive Reflection Test (CRT), Raven’s Progressive Matrices (MPR), Need for Cognition (NFC), and Faith in Intuition (FI) as predictors and participants’ absolute mean error as the criterion.

	B	SE B	β	*t*	*p*
CRT	−1.39	0.68	−0.24	−2.04	0.04 *
MPR	−0.80	0.58	−0.15	−1.36	0.17
NFC	1.02	0.35	0.31	2.86	0.00 **
FI	0.62	0.36	0.18	1.71	0.09

** < .01/* < .05.

## Data Availability

The data presented in this study are available on request from the corresponding author.

## Notes

1 The formula was coded using piped text, a feature in the Qualtrics platform (https://www.qualtrics.com, accessed on 1 May 2018) that allows previous answers to be used when presenting new problems. This feature allowed us to automatically make the base rates in each problem dependent on the estimated diagnosticity of the description in such a way that the Bayesian solutions were always 25%, 50%, or 75%.

2 On average, BRPs with the Bayesian solution P(A/C) = 0.50 are also conflict-problems given that base rates must oppose the diagnosticity of the descriptions in order to make the Bayesian solution converge on 50%. However, because 50% is closer to the range of values corresponding to the diagnosticity of the descriptions used in the BRP, these are not clear-cut examples of conflict problems. We thus refrained from classifying these BRPs as conflict problems.

## Appendix A

**Table A1:** Social groups and descriptions used in Base-Rate Problems.

Group A	Group B	Description	Mean Diagnosticity
18-year-old boys	18-year-old girls	Enjoys going out with friends, listening to music, and drinking beer.	51%
Italian girls	American girls	Is bilingual and enjoys reading and cooking in their free time.	62%
Buddhist girls	Muslim girls	Likes philosophy, has an aversion to material possessions, and uses second-hand clothing.	63%
Engineers	Lawyers	Enjoys reading science fiction and writing code on their computer.	69%
20-year-old men	50-year-old men	Enjoys techno music, wears jeans, dark glasses, has a small nose piercing, and enjoys dancing.	73%
People who buy clothes abroad	People who buy clothes at the mall	He lives on the top floor of one of the city’s best buildings and drives a Porsche.	73%

**Table A2:** CRT Problems.

Problem	Answer
1. A pencil and a notepad cost 1 euro and 10 cents. The notepad is 1 euro more expensive than the pencil. How much does the pencil cost?	5 cents
2. If 5 machines take 5 min to produce 5 pieces, how long do 60 machines take to make 60 pieces?	5 min
3. In a lake with water lilies everyday the number of water lilies doubles. It takes 48 days to fill the whole lake. How many days does it take to fill half the lake?	47 days
4. You have 5 coins. Your friend also has 5 coins. You give one coin to your friend. How many more coins does your friend now have in relation to you?	2 coins
5. You’re in a race and you outrun the person that is in second place. In what place are you now?	Second place
6. A brick weights a kilogram plus half a brick. How much does the brick weight?	2 kg

**Table A3:** Need For Cognition Scale.

Item
Thinking is not my idea of fun.
I try to anticipate and avoid situations where there is likely a chance I will have to think in depth about something.
I would prefer complex to simple problems.
I would prefer a task that is intellectual, difficult, and important to one that is somewhat important but does not require much thought.
I only think as hard as I have to.

**Table A4:** Faith in Intuition Scale.

Item
1. I trust my initial feelings about people.
2. I believe in trusting my hunches.
3. My initial impressions of people are almost always right.
4. When it comes to trusting people, I can usually rely on my “gut feelings”.
5. I can usually feel when a person is right or wrong even if I can’t explain how I know.
